# Evaluation of functioning among euthymic bipolar patients

**DOI:** 10.1192/j.eurpsy.2022.1057

**Published:** 2022-09-01

**Authors:** E. Mhiri, J. Ben Thabet, R. Feki, N. Smaoui, I. Gassara, L. Zouari, S. Omri, M. Maalej, N. Charfi, M. Maalej

**Affiliations:** Psychiatry C Department, Hedi Chaker, sfax, Tunisia

**Keywords:** functioning, impairment, bipolar, euthymic

## Abstract

**Introduction:**

Numerous studies have documented high rates of functional impairment among bipolar disorder patients, even during phases of euthymia.

**Objectives:**

To study different domains of functioning impairment in bipolar patients during euthymic phase.

**Methods:**

A cross-sectional and descriptive study of 78 patients followed for bipolar disorder, during euthymia, at the psychiatric outpatient clinic at CHU Hédi Chaker in Sfax. We used a socio-demographic and clinical data sheet and the Functioning Assessment Short Test (FAST) to assess functionning : A functional impairment was retained for a total FAST score > 11.

**Results:**

The average age was 36.27 years, the sex ratio was 5.5. Bipolar I disorder was diagnosed in 88.5% of patients. The mean age of onset was 27.73 years, and the mean duration of illness was 8.4 years. *The mean total score at the FAST was 22.23. *Functioning was altered on 69.2% of patients. *The occupational and the cognitive functioning were the two most altered domains in our population (respective mean scores : 8.69 and 5.74). *Autonomy was altered on 17.9% of patients. *Occupationnal functioning was altered on 76.9% of patients. *Cognitive functioning was altered on 70.5% of patients. *Financial issues were observed on 34.6% of patients. *Interpersonal relationships were altered on 41% of patients. *Leisure time difficulties were present with 24.4% of patients.

**Conclusions:**

This work has focused on the very high frequency of functional handicap in euthymic bipolar patients. Thus, several measures must be put in place to prevent or mitigate the negative effects of the impaired functioning on these patients.

**Disclosure:**

No significant relationships.

